# Loss of miR-369 Promotes Tau Phosphorylation by Targeting the Fyn and Serine/Threonine-Protein Kinase 2 Signaling Pathways in Alzheimer’s Disease Mice

**DOI:** 10.3389/fnagi.2019.00365

**Published:** 2020-01-31

**Authors:** Xiaoguang Yao, Xiaohui Xian, Mingxing Fang, Shujuan Fan, Wenbin Li

**Affiliations:** ^1^Department of Pathophysiology, Hebei Medical University, Shijiazhuang, China; ^2^Hebei Key Laboratory of Integrative Medicine on Liver-Kidney Patterns, Hebei University of Chinese Medicine, Shijiazhuang, China; ^3^Department of Surgery, Hebei University of Chinese Medicine, Shijiazhuang, China; ^4^Department of Intensive Care Medicine, Third Hospital of Hebei Medical University, Shijiazhuang, China; ^5^Neuroscience Research Center of Hebei Medical University, Shijiazhuang, China

**Keywords:** miR-369, tau protein, Fyn, serine/threonine-protein kinase 2, 3xTg Alzheimer’s disease mice

## Abstract

**Introduction:**

Alzheimer’s disease (AD) is a progressive neurodegenerative dementia with the key pathological hallmarks amyloid-beta deposition and neurofibrillary tangles composed of hyperphosphorylated tau. microRNAs (miRNAs) are small non-coding RNAs that contribute to the pathogenesis of AD. In this study, we investigated the effect of the loss of miR-369 on the phosphorylation of tau protein and the activation of the kinases Fyn and serine/threonine-protein kinase 2 (SRPK2) as the upstream molecules facilitating tau phosphorylation in miR-369 knockout 3xTg-AD mice.

**Methods:**

We generated miR-369 knockout 3xTg-AD mice and investigated their cognitive behaviors by maze tests. Real-time qPCR, western blot, and immunohistochemistry were performed to evaluate the expression of the miR-369 gene, phosphorylation of tau protein, and activation of Fyn and SRPK2. Luciferase reporter assays were applied to confirm the predicted targets of miR-369.

**Results:**

Knocking out miR-369 in 3xTg AD mice aggravated cognitive impairment, promoted hyperphosphorylation of tau, and upregulated Fyn and SRPK2. Restoring miR-369 reversed the hyperphosphorylation of tau and downregulated Fyn and SRPK2. Additionally, miR-369 was shown to target the 3′UTRs of Fyn and SRPK2 to regulate their expression levels.

**Conclusion:**

Loss of miR-369 promotes tau phosphorylation by targeting the Fyn and SRPK2 signaling pathways in AD mice, and supplementation with miR-369 might be a valuable option for AD therapeutic studies.

## Introduction

Alzheimer’s disease (AD) is one of the most common dementia diseases. The two major pathological hallmarks of AD are the presence of amyloid-beta (Aβ) plaques and neurofibrillary tangles induced by hyperphosphorylated tau protein. In recent years, an increasing number of studies have revealed that epigenetic factors including microRNAs (miRNAs) are involved in the aging process of the brain and AD pathogenesis by regulating gene expression, Aβ production, and tau phosphorylation. Previous studies have found that in mice, miR-369 is present in the developing forebrain at high levels and contributes to brain development by targeting Ncad expression. Moreover, miR-369 plays a role in neuronal differentiation and migration (Rago et al., 2014). Overexpression of miR-369 has been shown to inhibit glioblastoma cell proliferation (Shahar et al., 2016), while deficient expression of miR-369 might play a crucial role in the development of Hirschsprung disease (Pan et al., 2017). In addition, miR-369 is able to promote neuron differentiation (Winter, 2015). Several studies have reported that miR-369 is involved in AD pathogenesis. For example, Serpente et al. (2011) found that miR-369 may affect the occurrence and development of AD *via* the LDL receptor 1 gene (OLR1, rs1050283). Barak et al. (2013) found decreased levels of miR-369 in the hippocampal tissue of the 3xTg-AD mouse brain. Our preliminary experiment also showed a similar phenomenon in 6-month-old 3xTg-AD mice (see Supplementary Figure S1). Analysis of the GSE16759 dataset in the Gene Expression Omnibus (GEO) database showed that miR-369 decreases dramatically in AD samples (Nunez-Iglesias et al., 2010). Furthermore, miR-369 is a highly conserved “ancient” miRNA with 100% sequence identity among numerous species, including humans and mice. Therefore, to prove the role of miR-369 in AD pathogenesis, in the present study we applied 3xTg-AD mice with miR-369 knockout to investigate whether loss of miR-369 promotes phosphorylation of tau protein, the role of Fyn and serine/threonine-protein kinase 2 (SRPK2), which are kinases that promote phosphorylation of tau protein in AD (Lee et al., 2004; Hong et al., 2012), and affect cognitive behaviors, including Morris water maze (MWM) and Barnes maze tests. Furthermore, we investigated whether miR-369 can target Fyn and SRPK2 directly in cultured 293T cells using a luciferase reporter assay.

## Materials and Methods

### miR-369 Knockout Alzheimer’s Disease Mice

miR-369 KO 3xTg-AD mice (4 weeks old), regular C57/B6 mice (8 weeks old), and 3xTg-AD mice (8 weeks old) were purchased from The Experimental Animal Center of Beijing University of Medical Sciences (Beijing, China). 3xTg-AD mouse model is a typical model for AD that contains three mutations associated with familial AD (APP Swedish, MAPT P301L, and PSEN1 M146V) (Oddo et al., 2003). The mouse of this model develops Aβ deposits and tau pathology from 6 months and learning and memory deficits in the maze tests from about 6.5 months of age. In addition, the mouse expresses both Fyn and SRPK2 (Sterniczuk et al., 2010).

The miR-369 KO 3xTg-AD mice were obtained by hybridization of miR-369 KO and 3xTg-AD mice. Briefly, the targeting vector was designed to introduce LoxP sites on either side of the region encoding miR-369. A neomycin resistance cassette was included for positive selection and a TK cassette for negative selection. Gene targeting was carried out in embryonic stem (ES) cells derived from C57BL/6 mice using standard protocols. After the clones were identified by Southern blotting, targeted ES cells were injected into blastocysts to generate chimeric mice, which were in turn crossed with FLPe transgenic mice (C57BL/6) to delete the neomycin cassette, resulting in mice with a conditional allele for miR-369. The deletion of miR-369 was achieved by crossing these mice to the transgenic mice expressing Cre recombinase under a constitutive promoter. Following deletion, the mice were crossed away from the Cre transgene before experimental mice were generated. Routine genotyping of the mice was carried out by PCR using ear biopsy tissue. Then, the first-generation (F1) offspring gave double heterozygous mice, which were crossed with homozygous 3xTg-AD mice. Second-generation (F2) offspring (miR-369KO/AD mice, heterozygous for miR-369 and homozygous for 3xTg-AD, 12 months old) were used in the experiment (Supplementary Figure S2), and the regular 3xTg-AD mice were used as controls (10 mice/group, and male:female 1:1).

All mice were housed on a 12-h light/12-h dark cycle with access to food and water *ad libitum*. All animal care procedures and experiments were conducted according to the ARRIVE guidelines and approved by the Committee of Ethics on Animal Experiments of Hebei Medical University. All efforts were made to minimize the suffering and numbers of the animals used.

### Morris Water Maze Test

The Morris water maze (MWM) (Morris, 1984) was applied to investigate spatial learning and memory by measuring the time latency to find a hidden platform submerged in a pool. First, a large white circular pool was filled with water (21°C). Visual cues around the pool and a hidden platform in the pool were placed. On 4 consecutive days, the mice were placed into the water and allowed to swim and find the hidden platform within 60 s. Each animal underwent four trials with different starting directions. The paths of the mice were monitored until they reached the platform within 60 s. After that, to apply the probe test, the platform was removed from the pool, and then the mice were put back into water, and the time spent in the quadrant where the platform was previously located was recorded for a period within 60 s. A tracking camera device was placed to monitor behavioral experiments, and then the record was analyzed using video-tracking software.

### Barnes Maze Test

The Barnes maze (Anderson et al., 1998) test used in our study consisted of a circular platform (100 cm diameter) with 20 equally spaced holes (5 cm diameter) along the perimeter, which was placed 120 cm above the floor. One of the holes was set as the target for escape. Mice were placed in the center of the maze, facing a random direction, and then the mice were allowed to explore for 60 s. The time and the number of errors before reaching the target hole were recorded, and the path that the mice took was recorded as mentioned above.

### Intracerebroventricular Injection

The intracerebroventricular injection was performed with the following stereotactic coordinates: AP 0.5 mm, lateral 1 mm, and depth 2 mm (from the skull) (Jimenez-Mateos et al., 2011). Plasmid encoding miR-369 (0.2 nmol) or scrambled controls mixed with Lipofectamine 2000 (Invitrogen) dissolved in 2–3 μl artificial cerebrospinal fluid was injected slowly *via a* microsyringe. In total, three injections were performed with 1-month intervals. Three months after the injections, the mice were sacrificed, and brain tissues were collected for further investigation.

### Real-Time qPCR

RNA was extracted from the cerebral cortex using RNeasy mini kit and cDNA was generated using a reverse transcription kit (SuperScript III First-Strand Synthesis System; Invitrogen). Mature miR-369 was measured by TaqMan microRNA real-time qPCR (Thermo Fisher Scientific) using an ABI PRISM 7500 Real-Time qPCR System according to the manufacturer’s protocol. RNU43 was chosen as an internal control. Candidate gene expression was measured using a SYBR Green-based reagent (SYBR GreenER qPCR SuperMix for iCycler; Invitrogen) and a real-time qPCR system. The following primers were used: Fyn, forward 5′-CGAAGTTCAACACGGGGAGTA-3′ and reverse 5′-TGTATTCCAGAAGGCG AGCTT-3′; SRPK2, forward 5′-AG GACCCCGCAGATTACTG-3′ and reverse 5′-TTTCCCTTGC ATATCCCAGCA-3′; human microtubule-associated protein tau (MAPT), forward 5′-CCAAGTGT GGCTCATTAGGCA-3′ and reverse 5′-CCAATCTTCGACTGGACTCTGT-3′; and GAPDH, forward 5′-AATGGATTTGGACGCATTGGT-3′ and reverse 5′-TTTGCA CTGGTACGTGTTGAT-3′. All PCR analyses were performed in triplicate, and values were quantified with corresponding standard curves. miR-369 expression was normalized to that of RNU43, and the expression of Fyn and SRPK2 was normalized to GAPDH expression. Relative quantitation of gene expression was performed using the 2^–ΔΔCt^ method.

### Western Blotting

Cerebral cortex was lysed using RIPA buffer with a proteinase inhibitor. The samples were diluted to the same quantities (20 μg) and then loaded. Protein samples were separated by SDS-PAGE electrophoresis on 8–10% gels and then electro-transferred onto PVDF membranes (Millipore Corp). The membranes were blocked and then incubated with primary antibody (1:3,000) overnight at 4°C. Subsequently, the membrane was incubated with secondary antibodies (1:3,000 Abcam, anti-mouse IgG or anti-rabbit IgG). Then the blot was detected using the Western Bright ECL western blotting detection kit (Bio-Rad Laboratories). Equal sample loading was verified by the detection of GAPDH. The primary antibodies were as follows: mouse monoclonal anti-GAPDH (sc-66163, Santa Cruz, CA, United States), rabbit monoclonal anti-Fyn (ab125016, Abcam), rabbit monoclonal anti-SRPK2 (ab192238, Abcam), mouse monoclonal anti-tau (sc-390476, Santa Cruz, CA, United States), mouse monoclonal anti-tau (phospho Tyr18, 9G3, Genetex), and rabbit monoclonal anti- tau (phospho Ser214, ab170892, Abcam).

### Immunohistochemistry

Cerebral cortex sections (5 μm) on glass slides were deparaffinized and rehydrated. The sections were stained with antibodies (1:200 dilution) against Tyr18 for phosphorylation of tau, Fyn or SRPK2 and then incubated with a horseradish peroxidase-labeled dextran polymer coupled to an anti-mouse antibody. Staining that was clearly distinguishable from the background was considered positive. To evaluate the expression level, we quantified the OD values at 10 random area fields under 400 × magnification. Target protein expression was graded semiquantitatively according to the staining score results, and the mean values were used for statistical analysis.

### Cell Culture and Luciferase Reporter Assay

The 293T cells were purchased from the American type culture collection (ATCC) and were maintained in DMEM (HyClone), which were supplemented with 10% ultracentrifuged fetal bovine serum (FBS; Invitrogen), penicillin (100 U/ml; Invitrogen) and streptomycin (10 mg/ml; Invitrogen) at 37°C in a humidified atmosphere with 5% CO2. Trypsin (0.05%) and 0.02% ethylenediamine tetra-acetic acid (HyClone) were also used in the cell culture.

The 3′-untranslated region (UTR) of Fyn or SRPK2 containing binding sites for miR-369 was amplified by PCR with the following primers. Primers for Fyn: forward, 5′-CCGAACCTCCTCTGTGAACC-3′, and reverse, 5′-CATGGTA GCACCCACATGGT-3′. Primers for SRPK2: forward, 5′-CAC TGTGATCCTGGG GAAGG-3′, and reverse, 5′-CTGTGTGCAA CCTGCAAAGG-3′. A mutant 3′UTR of Fyn or SRPK2 was created using the QuikChange Site-Directed Mutagenesis Kit from Stratagene following the manufacturer’s instructions. The 3′UTR of Fyn or SRPK2 was cloned into the 3′UTR Luciferase/GFP reporter (Abcam) and then transfected into 293T cells with/without miR-369 cotransfection using Lipofectamine 2,000 reagent (Supplementary Data Sheet S1). Luciferase activity was measured using a Luciferase Assay System (Promega Corporation).

### ELISA

Soluble and insoluble Aβ40 and Aβ42 levels in the mice cortex were measured using human β-amyloid ELISA kits (Thermo Fisher Scientific). Homogenates were dissolved in TBS to collect soluble Aβ, and then the TBS was loaded directly into wells of plate. Also, the same quantity of homogenates was dissolved in guanidine to collect insoluble Aβ and then loaded in wells of plate after dilution at 1:100. The ELISAs were performed according to the manufacturer’s protocol, and the plates were read at 450 nm using a multi microplate reader.

### Statistics

All *in vitro* assays were in triplicates. Data were presented as the mean ± SEM. *P* values describing significance were based on *t* tests (2-tailed; α = 0.05) or analysis of repeated-measures two-way ANOVA. *P* values less than 0.05 indicate statistical significance.

## Results

### Knocking Out miR-369 Aggravated the Cognitive Impairment in Alzheimer’s Disease Mice

**FIGURE 1:**
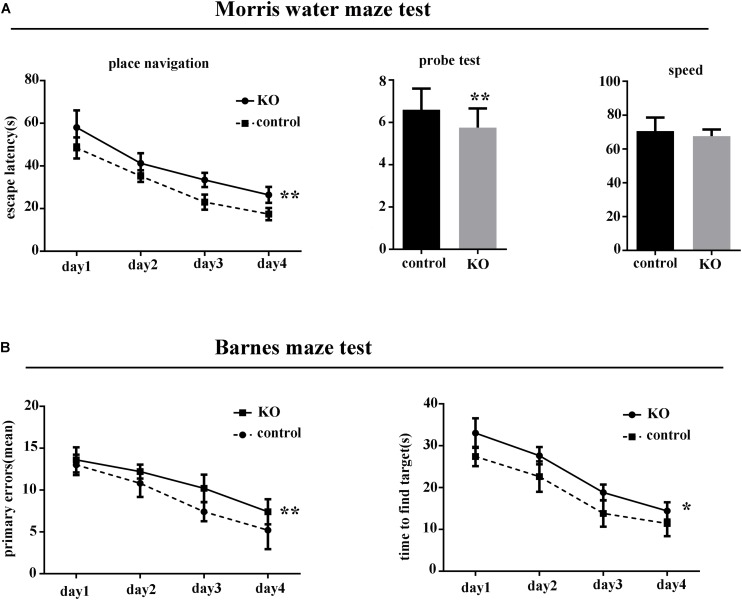
Knocking out miR-369 aggravated cognitive impairment in 3xTg-AD mice (12 months old, 10 mice/group, male:female = 1:1). **(A)** Indicates that the escape latencies significantly increased, and the number crossing the target platform significantly decreased after miR-369 knockout in 3xTg-AD mice in the Morris water maze (MWM) test. **(B)** Indicates more errors and more time to find the targets in the miR-369 knockout 3xTg-AD mice in the Barnes maze test. *p < 0.05 and **p < 0.01.

There was no difference between the knockout group and controls in body weight or body temperature (*p* > 0.05). However, in the MWM test, the latency to find the hidden platform was significantly increased in miR-369KO/AD mice compared with the control mice, and the performances of miR-369KO/AD mice were significantly poorer on the third and fourth days in the MWM test (Figure 1A). In the Barnes maze test, miR-369KO/AD mice had significantly more errors and took more time to find the targets in the trials compared with those observed for the controls (Figure 1B), which indicated that the learning ability of miR-369KO/AD mice had been impaired.

### Knocking Out miR-369 Promotes Hyperphosphorylation of Tau and Upregulation of Fyn and Serine/Threonine-Protein Kinase 2 in Alzheimer’s Disease Mice

**FIGURE 2:**
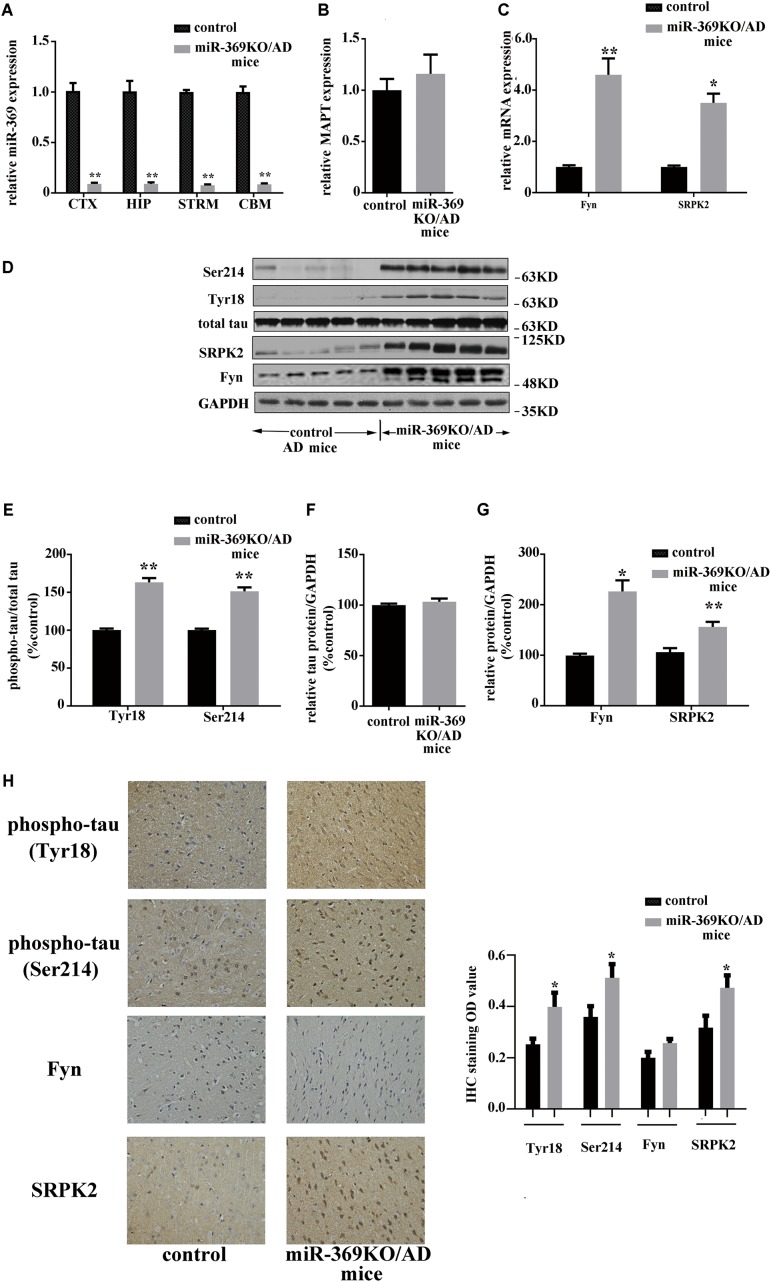
Changes in the levels of phosphorylated tau and the kinases Fyn and SRPK2 in miR-369KO/AD mice (12 months old, 10 mice/group, male:female = 1:1). **(A–C)** The real-time qPCR results indicating the loss of endogenous mature miR-369 expression in the brain regions (CTX, cortex; HIP, hippocampus; and STRM, striatum). **(A)** No change in MAPT mRNA, which encodes tau protein **(B),** and the upregulation of mRNA of Fyn and SRPK2 **(C)** in miR-369KO/AD mice. **(D)** Representative western blot images. **(E–G)** The quantitative presentations of the immunoblots. **(H)** The IHC staining results for tau protein, Fyn, and SRPK2 expression in the cerebral cortex. The results indicate an upregulation of phosphorylated tau protein (at Tyr18 and Ser214) and of Fyn and SRPK2 expression and no change in the total tau levels in miR-369KO/AD mice. *p < 0.05 and **p < 0.01.

Real-time qPCR analysis showed complete loss of mature (functional) miR-369 expression in the cortex, hippocampus, cerebellum, and striatum of miR-369KO/AD mice (Figure 2A). Western blotting analysis indicated that miR-369KO/AD mice had increased phosphorylation levels of tau protein at Ser214 and Tyr18 in the cerebral cortex, although both the MAPT gene (encoding tau protein) and total tau protein expression remained unchanged (Figures 2B,D–F). Moreover, the levels of both Fyn and SRPK2 were significantly increased in the miR-369KO/AD mice (Figures 2C,D,G). IHC indicated that knocking out miR-369 increased the phosphorylation level of tau protein in the cerebral cortex of AD mice. Moreover, the expression levels of SRPK2 and Fyn kinase were significantly upregulated in the miR-369KO/AD compared to controls (Figure 2H), although Fyn levels did not show statistical significance.

### The Expression of Amyloid-Beta, Apolipoprotein, and Glial Fibrillary Acidic Protein in miR-369 Knockout/Alzheimer’s Disease Mice

Insoluble extracellular Aβ deposits forming amyloid plaques and accumulation of ApoE and GFAP in the brain are all key histopathological hallmarks of AD (Braak and Braak, 1991). Moreover, soluble Aβ, particularly Aβ42 oligomers, are suspected to trigger neurotoxicity and synaptic dysfunctions (Lue et al., 1999). Therefore, both the soluble and insoluble forms of Aβ40 and Aβ42 were measured in the cortex of mice. The results showed no difference in Aβ40 and Aβ42 between 3xTg-AD mice with miR-369 KO compared to controls (Supplementary Figure S3). β-Site amyloid precursor protein cleaving enzyme 1 (BACE1) is an enzyme that is required for the production of Aβ. In this study, no changes in BACE1 protein expression were detected between the two mouse groups (Supplementary Figure S4).

ApoE is another protein involved in AD pathogenesis, so we evaluated ApoE expression in miR-369KO 3xTg-AD mice, and the data did not show a difference in ApoE expression between 3xTg-AD mice with and without miR-369KO. In addition, GFAP in astrocytes has been described as an index of reactive astrogliosis, a complex phenomenon closely related to neuronal damage seen in neurodegenerative diseases and other brain injuries (Cirillo et al., 2015). To assess glial activation, we measured GFAP in the cortex of the two groups of mice. No significant differences in GFAP expression were found in the 3xTg-AD mice with miR-369KO compared to the mice without KO.

### The Restoration of miR-369 Reverses the Hyperphosphorylation of Tau and the Upregulation of Fyn and Serine/Threonine-Protein Kinase 2 in miR-369 Knockout/Alzheimer’s Disease Mice

**FIGURE 3:**
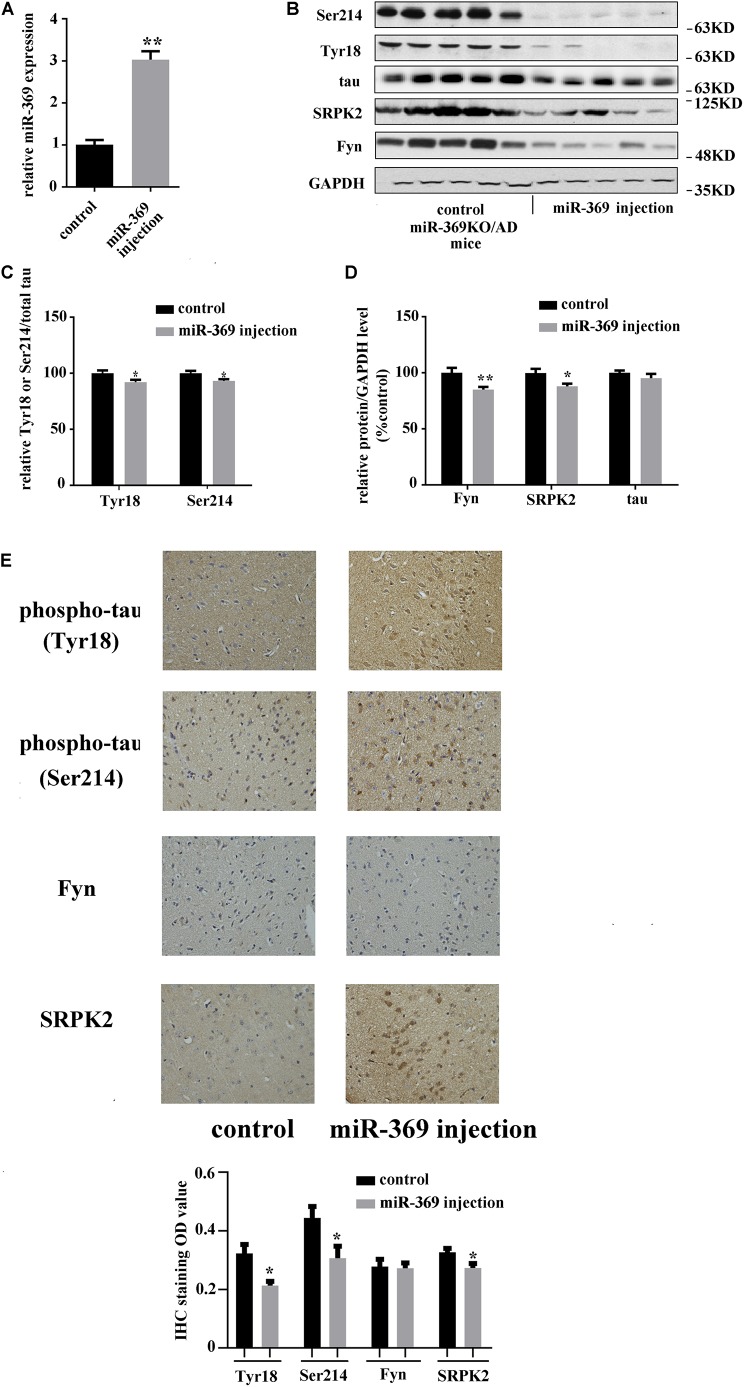
The effect of restoring miR-369 levels on tau hyperphosphorylation and upregulation of Fyn and SRPK2 in the cerebral cortex of miR-369KO/AD mice (10 mice/group, male:female = 1:1, treatments at 12 months old). **(A)** Real-time qPCR results indicating the restoration of mature miR-369 expression in the cerebral cortex after delivery of miR-369 in miR-369KO/AD mice. **(B–D)** Representative western blot images and the relative quantifications of the blots. **(E)** The results of IHC staining for phosphorylated and total tau proteins and the kinases Fyn and SRPK2, which show that restoring miR-369 significantly reversed the hyperphosphorylation of tau and the upregulation of Fyn and SRPK2 in the cerebral cortex of the mice. *p < 0.05 and **p < 0.01.

A total of 3 months after intracerebroventricular injection of miR-369 plasmid into miR-369KO/AD mice, cortex tissues were collected, and real-time qPCR results showed that miR-369 expression was significantly upregulated compared with that in control miR-369KO/AD mice (Figure 3A). Western blotting analysis indicated that supplementing miR-369 downregulated the expression level of Fyn and SRPK2 proteins and the phosphorylation level of tau protein at Ser214 and Tyr18 in the cerebral cortex and had no effect on total tau protein expression (Figures 3B–D). IHC showed significant downregulation in tau phosphorylation (at Ser214 and Tyr18) and Fyn and SRPK2 in miR-369-supplemented mice, although there was no statistically significant difference in Fyn levels between the two groups (Figure 3E).

### miR-369 Targets Fyn and Serine/Threonine-Protein Kinase 2

**FIGURE 4:**
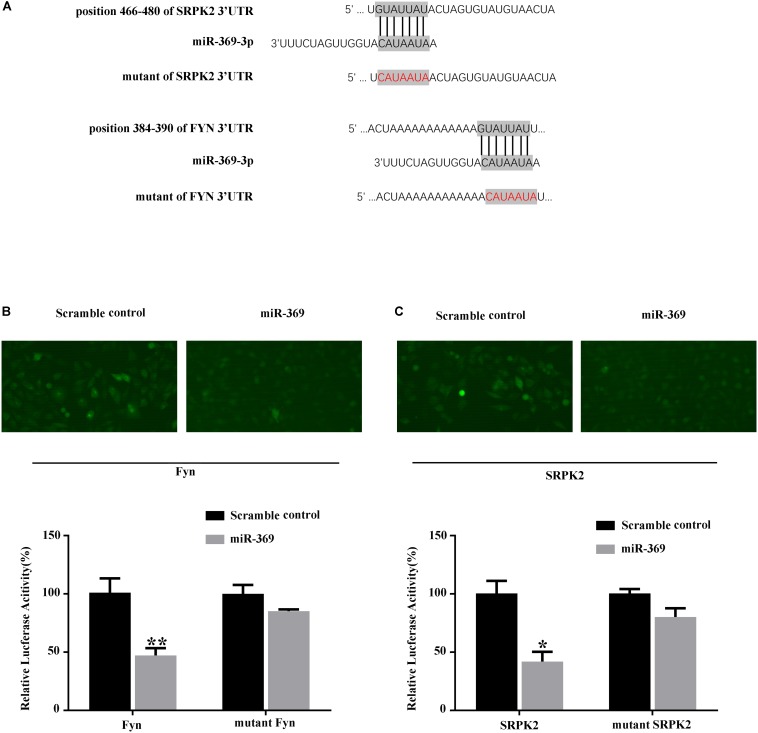
Luciferase assay shows the targets of miR-369. **(A)** Schematic diagram of the target sites of miR-369 on Fyn and SRPK2 mRNAs, which shows that miR-369 has potential complementary sites targeting the mouse Fyn and SRPK2 3′UTRs. **(B,C)** The results of the luciferase assay showing that miR-369 directly targeted the 3′UTRs of Fyn and SRPK2. All experiments were performed in triplicate. *p < 0.05 and **p < 0.01.

We first searched for miR-369 targets using algorithms based on the TargetScan website^1^ and found that both Fyn and SRPK2 could be targets of miR-369 (Figure 4A). Thus, we performed a luciferase assay in 293T cells by transfecting a reporter construct that contained the 3′UTR of mouse Fyn or SRPK2 mRNA containing the miR-369 binding site downstream of the luciferase or GFP gene. Subsequently, we cotransfected miR-369 or a scrambled control into 293T cells pre-containing reporter of Fyn or SRPK2 3′UTR and then measured luciferase activities. The results showed that miR-369 markedly reduced the luciferase activity of the FYN reporter construct. Results with the mutated mouse Fyn 3′UTR sequence cloned into the luciferase reporter construct showed that the reduced luciferase activity by miR-369 was abolished (Figure 4B). Similarly, miR-369 could target and inhibit luciferase activity when the 3′UTR of SRPK2 was cloned into a luciferase reporter (Figure 4C). Therefore, these findings demonstrate that miR-369 is able to target and bind both Fyn and SRPK2 and inhibit their expression levels.

## Discussion

Tau hyperphosphorylation plays a critical role in the pathogenesis of AD, which in turn restricts axonal transport, facilitates neurofibrillary tangle formation, and induces neuronal cell death. In the present study, we identified that loss of miR-369 contributes to tau phosphorylation *via* the upregulation of Fyn and SRPK2 activities, and restoring miR-369 levels might be a valuable option for AD therapeutic studies.

Over the years, expression profiling of miRNAs associated with AD has been performed in the brain tissues, cerebrospinal fluid, and blood of AD patients and transgenic AD mouse models. Although several miRNAs have been identified in AD, an understanding of the role of miRNA in human AD is still limited (Tan et al., 2013; Maoz et al., 2017; Quinlan et al., 2017). miRNAs may be involved in AD pathogenesis *via* multiple pathways, such as APP formation, phosphorylation of tau protein, and synaptic plasticity. For example, miR-138 can induce GSK-3β activation and tau phosphorylation in the brain (Wang et al., 2015). During aging in mice, miR-125b expression in the brain is increased, and increased miR-125b can regulate tau phosphorylation through MAPK signaling (Banzhaf-Strathmann et al., 2014). Therefore, by binding the 3′UTR regions of genes, miRNAs can contribute to translational repression or degradation of mRNAs of genes related to AD, which is a well-documented phenomenon known as transcriptional inhibition (Le Cam and Legraverend, 1995). In the present study, we focused on miR-369 because it is one of the most dramatically decreased miRNAs in AD patients and animal models. Furthermore, miR-369 is highly enriched in neural organs and is an “ancient” miRNA highly conserved among several species including humans and mice. We found that knocking out miR-369 expression aggravated the cognitive impairment of AD mice and increased the phosphorylation level of tau in these mice and that restoring miR-369 decreased the phosphorylation level of tau in miR-369KO/AD mice. Thus, it could be suggested that the loss of miR-369 could impair the cognitive ability of mice by increasing the phosphorylation level of tau, and restoring miR-369 might be a potential option for AD therapeutic studies.

A number of protein kinases are targeted by miR-369, which in turn regulate the phosphorylation level of tau. Through bioinformatic analysis with TargetScan, we identified multiple potential targets, and the kinases Fyn and SRPK2 were two of the most promising targets. Fyn is a Src-family non-receptor tyrosine kinase and has been shown to be correlated with hyperphosphorylation of tau protein in AD pathogenesis. For example, Fyn protein levels are increased in the brain tissue of AD patients, especially in the cerebellum and hippocampus (Zahratka et al., 2017), and the upregulation of Fyn increases the phosphorylation of tau protein on tyrosine-18 and promotes AD progression (Lee et al., 2004), whereas the inhibition of Fyn kinase activity can result in the improvement of synaptic function and memory in mice (Nygaard et al., 2014; Kaufman et al., 2015). In addition, there could exist an interaction between Aβ and Fyn. Amyloid protein can activate prion protein, which results in mGluR5 expression and an increase in Fyn activity, tau-Fyn interactions, and the phosphorylation level of NMDA receptors mediated by Fyn (Roberson et al., 2011; Larson et al., 2012; Um et al., 2012). SRPK2 is one of the cell cycle-regulated kinases that can phosphorylate serine residues of the serine-arginine-rich motif on substrates, which is enriched in the brain (Wang et al., 1999; Giannakouros et al., 2011). Some studies suggest that SRPK2 can contribute to AD development by promoting phosphorylation of tau protein. For example, Hong et al. (2012) found that SRPK2 facilitates the progression of AD by upregulating tau phosphorylation on Ser214. Wang et al. (2017) suggest that SRPK2 can phosphorylate δ-secretase on serine 226, and then the phosphorylated δ-secretase promotes AD-like pathology in the brain. In the present study, we investigated the effect of miR-369 loss on the kinases Fyn and SRPK2 and the correlation to tau phosphorylation. We found that miR-369 loss increased the phosphorylation level of tau *via* regulation of Fyn and SRPK2 in miR-369KO/AD mice, which could be reversed by miR-369 recovery. To examine the inhibitory effect of miR-369 on Fyn and SRPK2 expression, we transiently transfected the 3′UTR of Fyn or SRPK2 clone in a luciferase reporter and found that miR-369 directly inhibited reporter activities. It has been reported that Fyn has 3 isoforms (Rosenbloom et al., 2015), all of which share the same 3′UTR. Although we did not investigate the expression of these three isoforms individually, some studies have reported that FynT but not the other isoforms is activated in astrocytes upon treatment with amyloid peptides, instead of the other isoforms (Chun et al., 2004). We did not find that miR-369 can regulate tau protein expression directly. This is consistent with the search result that miR-369 has no bind site in the 3′UTR of the MAPT gene (encoding tau protein) in web-database of tragetscan (see text footnote 1) according to the sequences of miR-369 and MAPT gene indicated in NCBI. Therefore, these findings demonstrate that miR-369 is able to target and bind both Fyn and SRPK2 and to inhibit their expression levels, which in turn inhibit the phosphorylation of tau protein.

In summary, we demonstrated that loss of miR-369 promoted Fyn and SRPK2 expression and then increased the phosphorylation level of tau, while supplementation with miR-369 strongly suppressed the expression of both Fyn and SRPK2 and the phosphorylation level of tau in AD mice. These findings suggest that loss of miR-369 could be one of the factors contributing to AD through tau phosphorylation and that supplementation with miR-369 might be a potential option for AD therapeutic studies.

## Data Availability Statement

Publicly available datasets applied by this study can be found here: https://www.ncbi.nlm.nih.gov/geo/query/acc.cgi?acc=GSE16759.

## Ethics Statement

All animal care procedures and experiments were conducted according to the ARRIVE guidelines and approved by the Committee of Ethics on Animal Experiments of Hebei Medical University. All efforts were made to minimize the suffering and numbers of the animals used.

## Author Contributions

XY performed the certain experiments and wrote the manuscript. XY and XX completed the majority of the experiments. XY, XX, MF, and SF contributed to the idea and conception of this study. WL funded this research, supervised the experiments, and modified the manuscript. All authors read and approved the final manuscript.

## Conflict of Interest

The authors declare that the research was conducted in the absence of any commercial or financial relationships that could be construed as a potential conflict of interest.

## Footnotes

1 http://www.targetscan.org/

## Supplementary Material

FIGURE S1 The expression of miR-369 in 3xTg-AD mice and regular C57 mice during the aging process. It is shown that the expression level of miR-369 is decreased significantly in 3xTg-AD mice at 6-month age compared to 2 month age (*p* < 0.05), while no significant change in regular c57 mice (6 mice/group, male: female = 1:1).

FIGURE S2 Identification of miR-369KO/AD mice. **(A)** Positive results of APP/PS1/P301L (MAPT) transgenic mice genotype by RT-PCR. The RT-PCR product bands of APP gene (350 bp) and PS1 gene (608 bp) are identified clearly. **(B)** Targeting strategy. The structures of the wild-type allele and the disrupted allele are shown. **(C)** Correctly targeted ES cells were used to generate a conditional miR-369 allele in mice using standard techniques and verified by southern blot. Mice were crossed to Flp transgenic mice to excise Neomycin resistance cassette and then Cre expressing mice to delete miR-369. **(D)** Representative images of Southern blotting analysis with 3′ external probes specific for the WT (arrows) and mutant alleles (arrowheads) of miR-369 clusters.

FIGURE S3 Soluble and insoluble Aβ peptides in the cerebral cortex of miR-369KO/AD mice. Aβ quantification was performed by ELISA, and there is no significant difference in level of soluble and insoluble Aβ between miR-369 knockout 3xTg-AD mice and controls (6 mice/group, male: female = 1:1).

FIGURE S4 Changes in the levels of ApoE, BACE1, and GFAP in cerebral cortex of miR-369KO/AD mice (representative images of western blot, and the quantitative presentation of the immunoblot). All of results indicate that no significant changes are detected in expression of ApoE, BACE1, and GFAP. **p* < 0.05 and ***p* < 0.01 (6 mice/group, male: female = 1:1).

DATA SHEET S1 Vector map and their sequence.
