# Supplementary figures and images for: Loss of miR-369 Promotes Tau Phosphorylation by Targeting the Fyn and Serine/Threonine-Protein Kinase 2 Signaling Pathways in Alzheimer’s Disease Mice
Source: Front Aging Neurosci. 2020 Jan 31;11:365. doi: 10.3389/fnagi.2019.00365 (PMC7004974; doi:10.3389/fnagi.2019.00365)

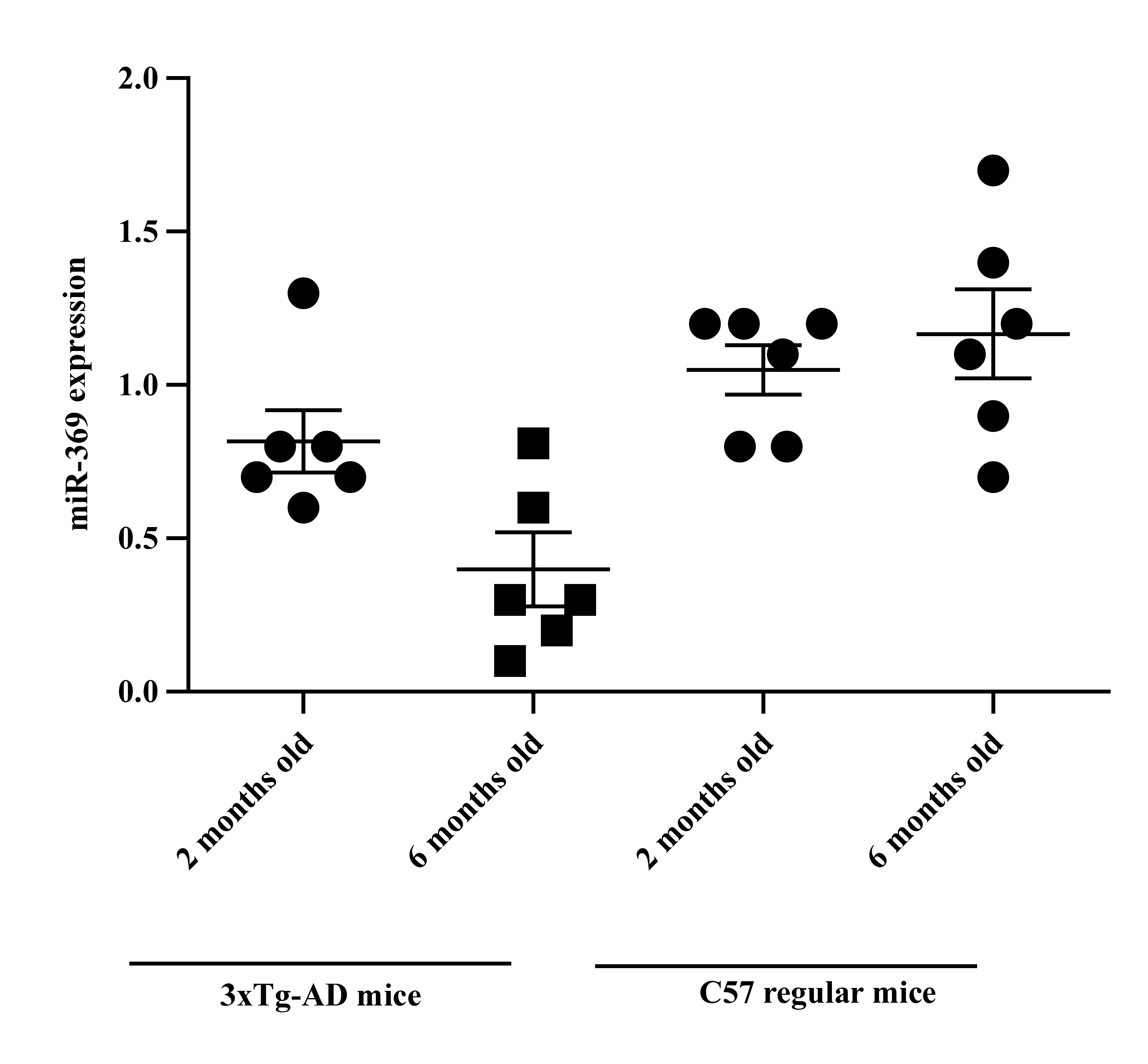

Supplement: FIGURE S1 — The expression of miR-369 in 3xTg-AD mice and regular C57 mice during the aging process. It is shown that the expression level of miR-369 is decreased significantly in 3xTg-AD mice at 6-month age compared to 2 month age (p < 0.05), while no significant change in regular c57 mice (6 mice/group, male: female = 1:1). [file Image_1.TIF]

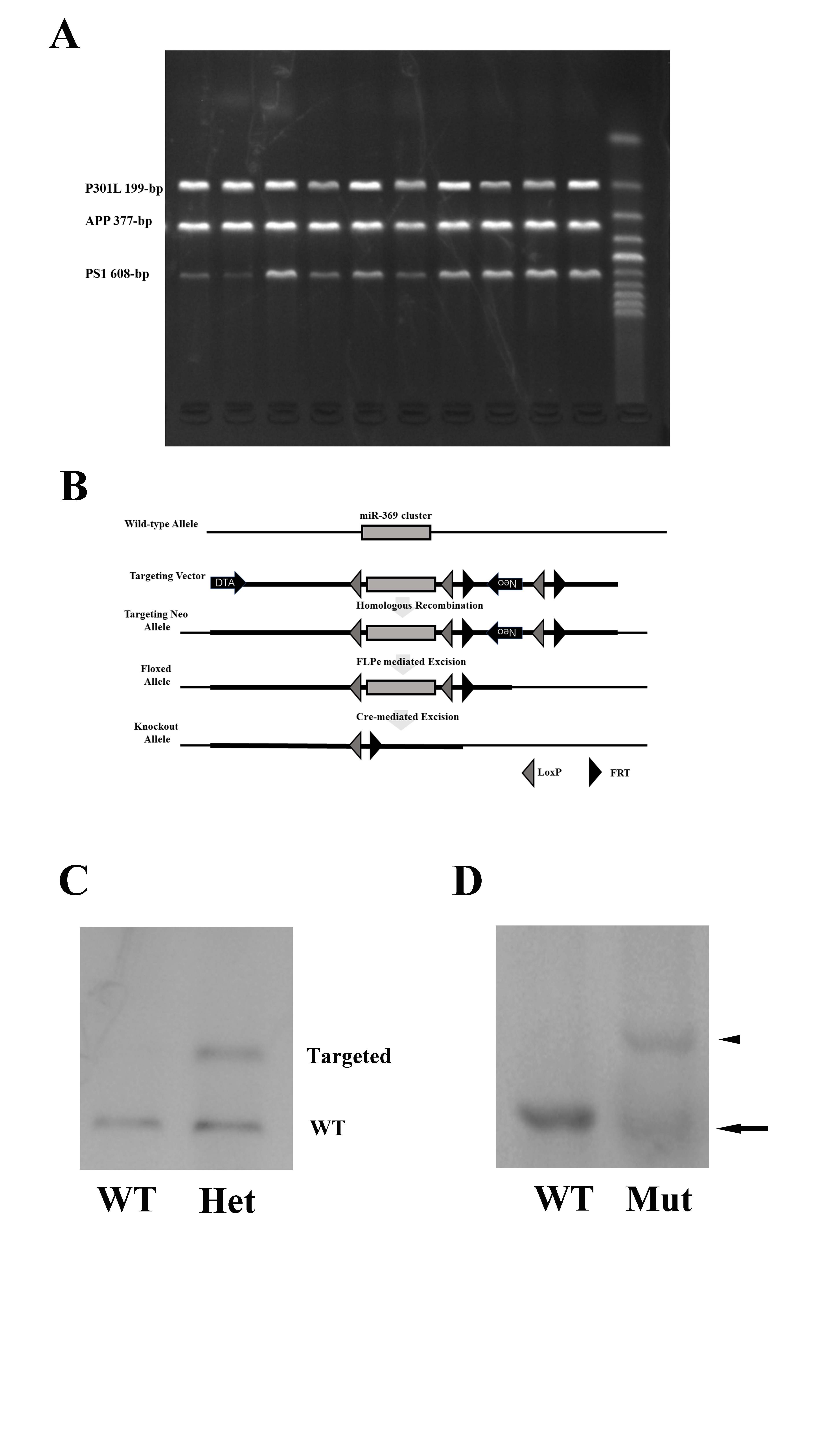

Supplement: FIGURE S2 — Identification of miR-369KO/AD mice. (A) Positive results of APP/PS1/P301L (MAPT) transgenic mice genotype by RT-PCR. The RT-PCR product bands of APP gene (350 bp) and PS1 gene (608 bp) are identified clearly. (B) Targeting strategy. The structures of the wild-type allele and the disrupted allele are shown. (C) Correctly targeted ES cells were used to generate a conditional miR-369 allele in mice using standard techniques and verified by southern blot. Mice were crossed to Flp transgenic mice to excise Neomycin resistance cassette and then Cre expressing mice to delete miR-369. (D) Representative images of Southern blotting analysis with 3′ external probes specific for the WT (arrows) and mutant alleles (arrowheads) of miR-369 clusters. [file Image_2.TIF]

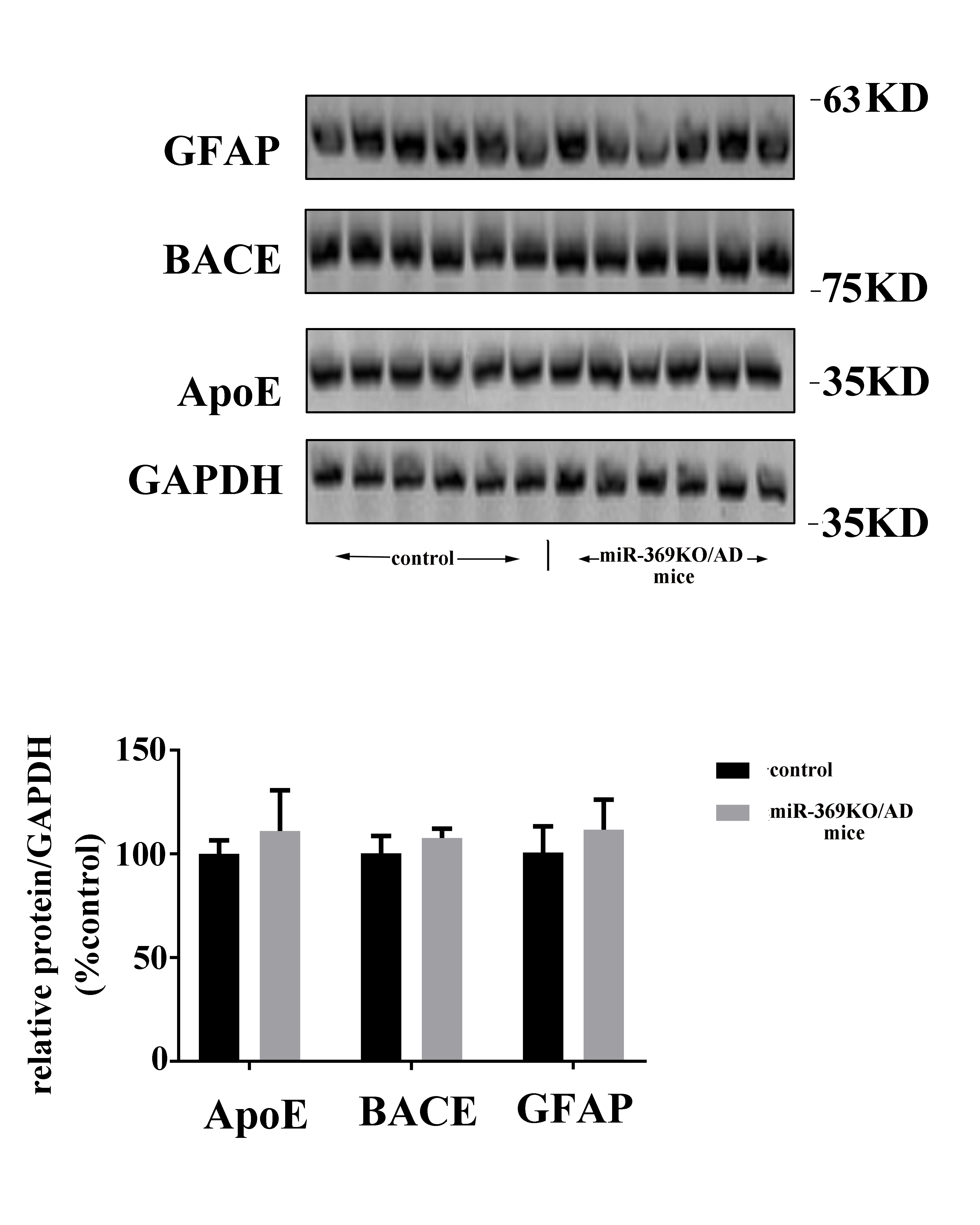

Supplement: FIGURE S4 — Changes in the levels of ApoE, BACE1, and GFAP in cerebral cortex of miR-369KO/AD mice (representative images of western blot, and the quantitative presentation of the immunoblot). All of results indicate that no significant changes are detected in expression of ApoE, BACE1, and GFAP. *p < 0.05 and **p < 0.01 (6 mice/group, male: female = 1:1). [file Image_4.TIF]
