# Supplementary material for: Loss of miR-369 Promotes Tau Phosphorylation by Targeting the Fyn and Serine/Threonine-Protein Kinase 2 Signaling Pathways in Alzheimer’s Disease Mice
Source: Front Aging Neurosci. 2020 Jan 31;11:365. doi: 10.3389/fnagi.2019.00365 (PMC7004974; doi:10.3389/fnagi.2019.00365)
Supplement: DATA SHEET S1 — Vector map and their sequence. [file Data_Sheet_1.PDF]

pLenti-UTR-Luc:

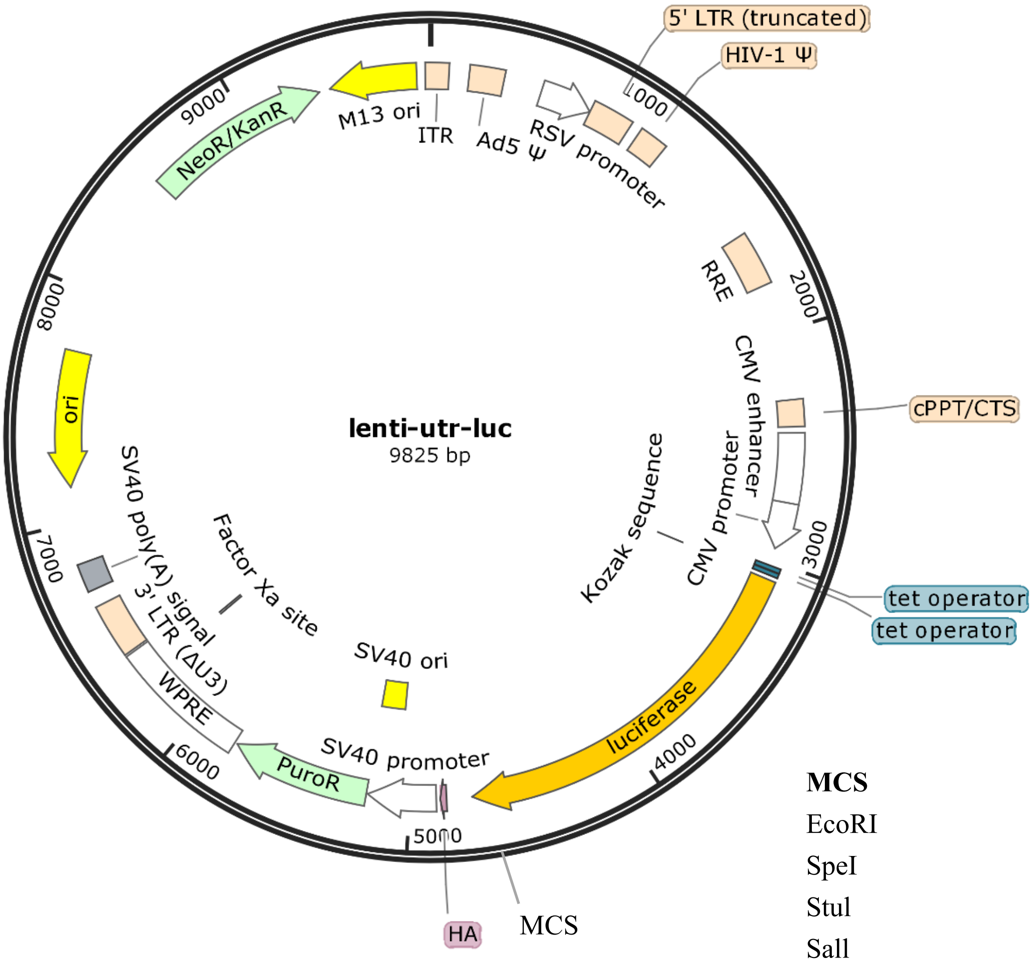

MCS

EcoRI

SpeI

StuI

SalI

EcoRV

NotI

BamHI

XbaI

XhoI

Sequence of pLenti-UTR-Luc:

ttttggattgaagccaatatgataatgagggggtggagtttgtgacgtggcgcggggcgtgggaacggggcgggtgacgtagtagtgtggc
ggaagtgtgatgttgcaagtgtggcggaacacatgtaagcgacggatgtggcaaaagtgacgtttttggtgtgcgccggtgtacacaggaag
tgacaattttcgcgcggttttaggcggatgttgtagtaaatttgggcgtaaccgagtaagatttggccattttcgcgggaaaactgaataagagg
aagtgaaatctgaataattttgtgttactcatagcgcgtaatacggcagacctcagcgctagattattgaagcatttatcagggttattgtctcatga
gcggatacatatttgaatgtatttagaaaaataaacaaataggggttccgcgcacatttccccgaaaagtgccacctgacgttaactataacggt
cctaaggtagcgaaaatgtagtcttatgcaatactcttgtagtcttgcaacatggtaacgatgagttagcaacatgccttacaaggagagaaaaa
gcaccgtgcatgccgattggtggaagtaaggtggtacgatcgtgccttattaggaaggcaacagacgggtctgacatggattggacgaacca
ctgaattgccgcattgcagagatattgtatttaagtgcctagctcgatacataaacgggtctctctggttagaccagatctgagcctgggagctct
ctggctaactagggaacccactgcttaagcctcaataaagcttgccttgagtgcttcaagtagtgtgtgcccgtctgttgtgtgactctggtaact

agagatccctcagacccttttagtcagtgtggaaaatctctagcagtggcgcccgaacagggacttgaaagcgaaagggaaaccagaggag
ctctctcgacgcaggactcggcttgctgaagcgcgcacggcaagaggcgaggggcggcgactggtgagtacgccaaaaattttgactagc
ggaggctagaaggagagagatgggtgcgagagcgtcagtattaagcgggggagaattagatcgcgatgggaaaaaattcggttaaggcca
gggggaaagaaaaaatataaattaaaacatatagtatgggcaagcagggagctagaacgattcgcagttaatcctggcctgttagaaacatca
gaaggctgtagacaaatactgggacagctacaaccatcccttcagacaggatcagaagaacttagatcattatataatacagtagcaaccctct
attgtgtgcatcaaaggatagagataaaagacaccaaggaagctttagacaagatagaggaagagcaaaacaaaagtaagaccaccgcac
agcaagcccgctgatcttcagacctggaggaggagatatgagggacattggagaagtgaattatataaatataaagtagtaaaaattgaacca
ttaggagtagcacccaccaaggcaaagagaagagtggtgcagagagaaaaaagagcagtgggaataggagctttgttccttgggttcttgg
gagcagcaggaagcactatgggcgcagcgtcaatgacgctgacggtacaggccagacaattattgtctggtatagtgcagcagcagaacaa
tttgctgagggctattgaggcgcaacagcatctgttgcaactcacagtctggggcatcaagcagctccaggcaagaatcctggctgtggaaag
atacctaaaggatcaacagctcctggggatttggggttgctctggaaaactcatttgcaccactgctgtgccttggaatgctagttggagtaataa
atctctggaacagatttggaatcacacgacctggatggagtgggacagagaaattaacaattacacaagcttaatacactccttaattgaagaat
cgcaaaaccagcaagaaaagaatgaacaagaattattggaattagataaatgggcaagtttgtggaattggtttaacataacaaattggctgtg
gtatataaaattattcataatgatagtaggaggcttggtaggtttaagaatagtttttgctgtactttctatagtgaatagagttaggcagggatattc
accattatcgtttcagacccacctcccaaccccgaggggacccgacaggcccgaaggaatagaagaagaaggtggagagagagacagag
acagatccattcgattagtgaacggatctcgacggtatcgaaagcttgggattcgaatttaaaagaaaaggggggattggggggtacagtgca
ggggaaagaatagtagacataatagcaacagacatacaaactaaagaactacaaaaacaaattacaaaaattcaaaattttcgggtttttcgaa
cctagggttccgcgttacataacttacggtaaatggcccgcctggctgaccgcccaacgacccccgcccattgacgtcaataatgacgtatgtt
cccatagtaacgccaatagggactttccattgacgtcaatgggtggagtatttacggtaaactgcccacttggcagtacatcaagtgtatcatatg
ccaagtacgccccctattgacgtcaatgacggtaaatggcccgcctggcattatgcccagtacatgaccttatgggactttcctacttggcagta
catctacgtttagtcatcgctattaccatggtgatgcggttttggcagtacatcaatgggcgtggatagcggtttgactcacggggatttccaagt
ctccaccccattgacgtcaatgggagtttgttttggcaccaaaatcaacgggactttccaaaatgtcgtaacaactccgccccattgacgcaaat
gggcggtaggcgtgtacggtgggaggtctatataagcagagctcgtttagtgaaccgtcagatcgcctggagacgccatccacgctgttttga
cctccatagaagaaccgagtttaaactccctatcagtgatagagatctccctatcagtgatagagagctagagctagcagccaccatggaagat
gccaaaaacattaagaagggcccagcgccattctacccactcgaagacgggaccgccggcgagcagctgcacaaagccatgaagcgcta
cgccctggtgcccggcaccatcgcctttaccgacgcacatatcgaggtggacattacctacgccgagtacttcgagatgagcgttcggctgg
cagaagctatgaagcgctatgggctgaatacaaaccatcggatcgtggtgtgcagcgagaatagcttgcagttcttcatgcccgtgttgggtgc
cctgttcatcggtgtggctgtggccccagctaacgacatctacaacgagcgcgagctgctgaacagcatgggcatcagccagcccaccgtc
gtattcgtgagcaagaaagggctgcaaaagatcctcaacgtgcaaaagaagctaccgatcatacaaaagatcatcatcatggatagcaagac
cgactaccagggcttccaaagcatgtacaccttcgtgacttcccatttgccacccggcttcaacgagtacgacttcgtgcccgagagcttcgac
cgggacaaaaccatcgccctgatcatgaacagtagtggcagtaccggattgcccaagggcgtagccctaccgcaccgcaccgcttgtgtcc
gattcagtcatgcccgcgaccccatcttcggcaaccagatcatccccgacaccgctatcctcagcgtggtgccatttcaccacggcttcggcat
gttcaccacgctgggctacttgatctgcggctttcgggtcgtgctcatgtaccgcttcgaggaggagctattcttgcgcagcttgcaagactata
agattcaatctgccctgctggtgcccacactatttagcttcttcgctaagagcactctcatcgacaagtacgacctaagcaacttgcacgagatc
gccagcggcggggcgccgctcagcaaggaggtaggtgaggccgtggccaaacgcttccacctaccaggcatccgccagggctacggcc
tgacagaaacaaccagcgccattctgatcacccccgaaggggacgacaagcctggcgcagtaggcaaggtggtgcccttcttcgaggctaa
ggtggtggacttggacaccggtaagacactgggtgtgaaccagcgcggcgagctgtgcgtccgtggccccatgatcatgagcggctacgtt
aacaaccccgaggctacaaacgctctcatcgacaaggacggctggctgcacagcggcgacatcgcctactgggacgaggacgagcacttc
ttcatcgtggaccggctgaagagcctgatcaaatacaagggctaccaggtagccccagccgaactggagagcatcctgctgcaacacccca
acatcttcgacgccggggtcgccggcctgcccgacgacgatgccggcgagctgcccgccgcagtcgtcgtgctggaacacggtaaaacc
atgaccgagaaggagatcgtggactatgtggccagccaggttacaaccgccaagaagctgcgcggtggtgttgtgttcgtggacgaggtgc
ctaaaggactgaccggcaagttggacgcccgcaagatccgcgagattctcattaaggccaagaagggcggcaagatcgccgtgcaattgta
aggtaccccagtgtggtggcctgcaggtgaattcactagtaccggtaggcctgtcgacgatatcgggcccgcggccgctggatcctctagac
tgcagctcgagtacccatacgacgtcccagactacgcttgagtttaaacacgcgtggtgtggaaagtccccaggctccccagcaggcagaa

gtatgcaaagcatgcatctcaattagtcagcaaccaggtgtggaaagtccccaggctccccagcaggcagaagtatgcaaagcatgcatctc
aattagtcagcaaccatagtcccgcccctaactccgcccatcccgcccctaactccgcccagttccgcccattctccgccccatggctgactaa
ttttttttatttatgcagaggccgaggccgcctcggcctctgagctattccagaagtagtgaggaggcttttttggaggccatgaccgagtacaag
cccacggtgcgcctcgccacccgcgacgacgtccctcgggccgtacgcaccctcgccgccgcgttcgccgactaccccgccacgcgcca
caccgtggacccggaccgccacatcgagcgggtcaccgagctgcaagaactcttcctcacgcgcgtcgggctcgacatcggcaaggtgtg
ggtcgcggacgacggcgccgcggtggcggtctggaccacgccggagagcgtcgaagcgggggcggtgttcgccgagatcggcccgcg
catggccgagttgagcggttcccggctggccgcgcagcaacagatggaagggctcctggcgccgcaccggcccaaggagcccgcgtgg
ttcctggccaccgtcggcgtctcgcccgaccaccagggcaagggtctgggcagcgccgtcgtgctccccggagtggaggcggccgagcg
cgccggggtgcccgccttcctggagacctccgcgccccgcaacctccccttctacgagcggctcggcttcaccgtcaccgccgacgtcgag
gtgcccgaaggaccgcgcacctggtgcatgacccgcaagcccggtgcctgaacgcgttccggaaatcaacctctggattacaaaatttgtga
aagattgactggtattcttaactatgttgctccttttacgctatgtggatacgctgctttaatgcctttgtatcatgctattgcttcccgtatggctttcatt
ttctcctccttgtataaatcctggttgctgtctctttatgaggagttgtggcccgttgtcaggcaacgtggcgtggtgtgcactgtgtttgctgacgc
aacccccactggttggggcattgccaccacctgtcagctcctttccgggactttcgctttccccctccctattgccacggcggaactcatcgccg
cctgccttgcccgctgctggacaggggctcggctgttgggcactgacaattccgtggtgttgtcggggaagctgacgtcctttccatggctgct
cgcctgtgttgccacctggattctgcgcgggacgtccttctgctacgtcccttcggccctcaatccagcggaccttccttcccgcggcctgctgc
cggctctgcggcctcttccgcgtctcgccttcgccctcagacgagtcggatctccctttgggccgcctccccgcctgtccggatggaagggct
aattcactcccaacgaatacaagatctgctttttgcttgtactgggtctctctggttagaccagatctgagcctgggagctctctggctaactaggg
aacccactgcttaagcctcaataaagcttgccttgagtgcttcaagtagtgtgtgcccgtctgttgtgtgactctggtaactagagatccctcaga
cccttttagtcagtgtggaaaatctctagcagtagtagttcatgtcatcttattattcagtatttataacttgcaaagaaatgaatatcagagagtgag
aggaacttgtttattgcagcttataatggttacaaataaagcaatagcatcacaaatttcacaaataaagcatttttttcactgcattctagttgtggttt
gtccaaactcatcaatgtatcttatcatgtctggcatctatgtcgggtgcggagaaagaggtaatgaaatggcattatgggtattatgggtctgcat
taatgaatcggccaacgatcccggtgtgaaataccgcacagatgcgtaaggagaaaataccgcatcaggcgctcttccgcttcctcgctcact
gactcgctgcgctcggtcgttcggctgcggcgagcggtatcagctcactcaaaggcggtaatacggttatccacagaatcaggggataacgc
aggaaagaacatgtgagcaaaaggccagcaaaaggccaggaaccgtaaaaaggccgcgttgctggcgtttttccataggctccgcccccct
gacgagcatcacaaaaatcgacgctcaagtcagaggtggcgaaacccgacaggactataaagataccaggcgtttccccctggaagctccc
tcgtgcgctctcctgttccgaccctgccgcttaccggatacctgtccgcctttctcccttcgggaagcgtggcgctttctcatagctcacgctgta
ggtatctcagttcggtgtaggtcgttcgctccaagctgggctgtgtgcacgaaccccccgttcagcccgaccgctgcgccttatccggtaacta
tcgtcttgagtccaacccggtaagacacgacttatcgccactggcagcagccactggtaacaggattagcagagcgaggtatgtaggcggtg
ctacagagttcttgaagtggtggcctaactacggctacactagaaggacagtatttggtatctgcgctctgctgaagccagttaccttcggaaaa
agagttggtagctcttgatccggcaaacaaaccaccgctggtagcggtggtttttttgtttgcaagcagcagattacgcgcagaaaaaaaggat
ctcaagaagatcctttgatcttttctacggggtctgacgctcagtggaacgaaaactcacgttaagggattttggtcatgagattatcaaaaagga
tcttcacctagatccttttaaattaaaaatgaagttttaaatcaatctaaagtatatatgagtaaacttggtctgacagttaccaatgcttaatcagtga
ggcacctatctcagcgatctgtctatttcgttcatccatagttgcctgactccccgtcgtgtagataactacgatacgggagggcttaccatctgg
ccccagtgctgcaatgataccgcgagacccacgctcaccggctccagatttatcagcaataaaccagccagccggaagggccgagcgcag
aagtggtcctgcaactttatccgcctccatccagtctattaattgttgccgggaagctagagtaagtagttcgccagttaatagtttgcgcaacgtt
gttgaaaaaggatcttcacctagatccttttcacgtagaaagccagtccgcagaaacggtgctgaccccggatgaatgtcagctactgggctat
ctggacaagggaaaacgcaagcgcaaagagaaagcaggtagcttgcagtgggcttacatggcgatagctagactgggcggttttatggaca
gcaagcgaaccggaattgccagctggggcgccctctggtaaggttgggaagccctgcaaagtaaactggatggctttctcgccgccaagga
tctgatggcgcaggggatcaagctctgatcaagagacaggatgaggatcgtttcgcatgattgaacaagatggattgcacgcaggttctccgg
ccgcttgggtggagaggctattcggctatgactgggcacaacagacaatcggctgctctgatgccgccgtgttccggctgtcagcgcagggg
cgcccggttctttttgtcaagaccgacctgtccggtgccctgaatgaactgcaagacgaggcagcgcggctatcgtggctggccacgacggg
cgttccttgcgcagctgtgctcgacgttgtcactgaagcgggaagggactggctgctattgggcgaagtgccggggcaggatctcctgtcatc
tcaccttgctcctgccgagaaagtatccatcatggctgatgcaatgcggcggctgcatacgcttgatccggctacctgcccattcgaccaccaa
gcgaaacatcgcatcgagcgagcacgtactcggatggaagccggtcttgtcgatcaggatgatctggacgaagagcatcaggggctcgcg

ccagccgaactgttcgccaggctcaaggcgagcatgcccgacggcgaggatctcgtcgtgacccatggcgatgcctgcttgccgaatatcat
ggtggaaaatggccgcttttctggattcatcgactgtggccggctgggtgtggcggaccgctatcaggacatagcgttggctacccgtgatatt
gctgaagagcttggcggcgaatgggctgaccgcttcctcgtgctttacggtatcgccgctcccgattcgcagcgcatcgccttctatcgccttct
tgacgagttcttctgaattttgttaaaatttttgttaaatcagctcatttttaaccaataggccgaaatcggcaacatcccttataaatcaaaagaata
gaccgcgatagggttgagtgttgttccagtttggaacaagagtccactattaaagaacgtggactccaacgtcaaagggcgaaaaaccgtcta
tcagggcgatggcccactacgtgaaccatcacccaaatcaagttttttgcggtcgaggtgccgtaaagctctaaatcggaaccctaaagggag
cccccgatttagagcttgacggggaaagccggcgaacgtggcgagaaaggaagggaagaaagcgaaaggagcgggcgctagggcgct
ggcaagtgtagcggtcacgctgcgcgtaaccaccacacccgcgcgcttaatgcgccgctacagggcgcgtccattcgccattcaggatcga
attaattcttaattaacatcatcaataatatacctt

pLenti-UTR-GFP:

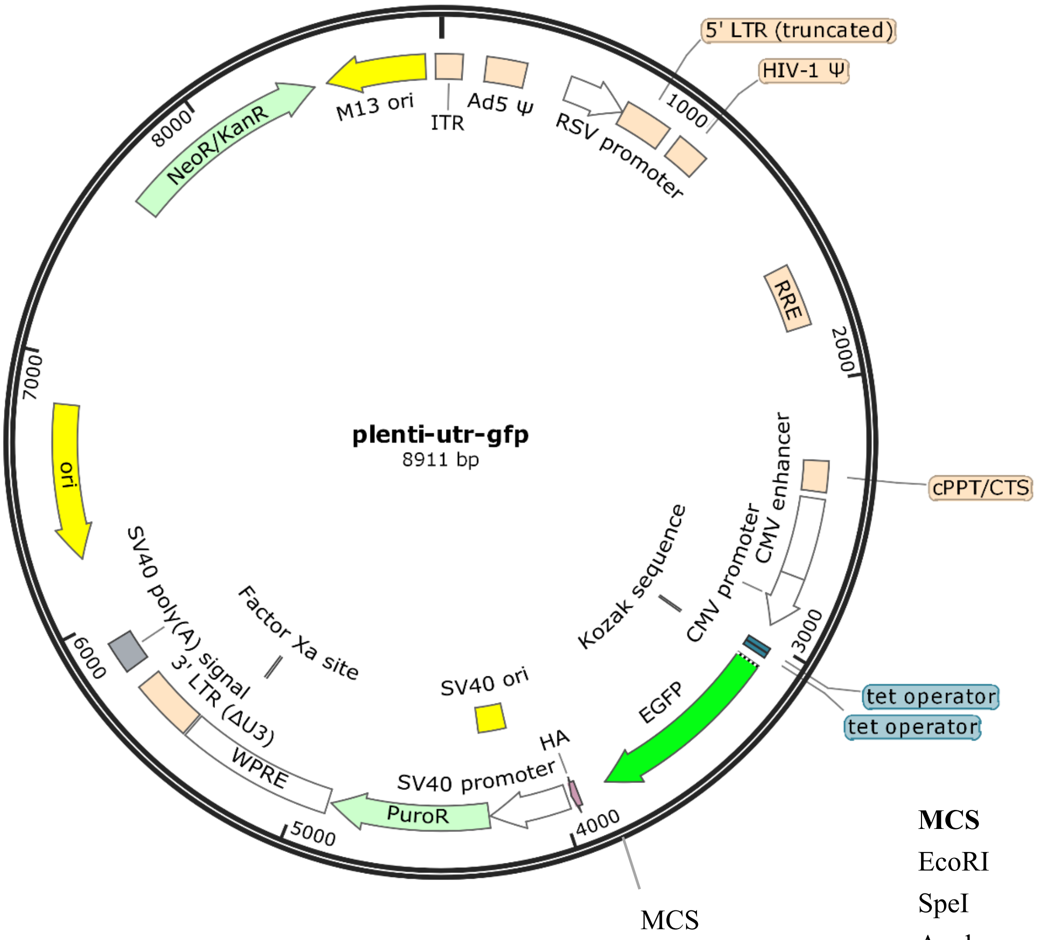

MCS

EcoRI

SpeI

AgeI

StuI

SalI

EcoRV

ApaI

NotI

BamHI

XbaI

XhoI

Sequence of pLenti-UTR-GFP:

ttttggattgaagccaatatgataatgagggggtggagtttgtgacgtggcgcggggcgtgggaacggggcgggtgacgtagtagtgtggc
ggaagtgtgatgttgcaagtgtggcggaacacatgtaagcgacggatgtggcaaaagtgacgtttttggtgtgcgccggtgtacacaggaag
tgacaattttcgcgcggttttaggcggatgttgtagtaaatttgggcgtaaccgagtaagatttggccattttcgcgggaaaactgaataagagg
aagtgaaatctgaataattttgtgttactcatagcgcgtaatacggcagacctcagcgctagattattgaagcatttatcagggttattgtctcatga
gcggatacatatttgaatgtatttagaaaaataaacaaataggggttccgcgcacatttccccgaaaagtgccacctgacgttaactataacggt
cctaaggtagcgaaaatgtagtcttatgcaatactcttgtagtcttgcaacatggtaacgatgagttagcaacatgccttacaaggagagaaaaa
gcaccgtgcatgccgattggtggaagtaaggtggtacgatcgtgccttattaggaaggcaacagacgggtctgacatggattggacgaacca
ctgaattgccgcattgcagagatattgtatttaagtgcctagctcgatacataaacgggtctctctggttagaccagatctgagcctgggagctct
ctggctaactagggaacccactgcttaagcctcaataaagcttgccttgagtgcttcaagtagtgtgtgcccgtctgttgtgtgactctggtaact
agagatccctcagacccttttagtcagtgtggaaaatctctagcagtggcgcccgaacagggacttgaaagcgaaagggaaaccagaggag
ctctctcgacgcaggactcggcttgctgaagcgcgcacggcaagaggcgaggggcggcgactggtgagtacgccaaaaattttgactagc
ggaggctagaaggagagagatgggtgcgagagcgtcagtattaagcgggggagaattagatcgcgatgggaaaaaattcggttaaggcca
gggggaaagaaaaaatataaattaaaacatatagtatgggcaagcagggagctagaacgattcgcagttaatcctggcctgttagaaacatca
gaaggctgtagacaaatactgggacagctacaaccatcccttcagacaggatcagaagaacttagatcattatataatacagtagcaaccctct
attgtgtgcatcaaaggatagagataaaagacaccaaggaagctttagacaagatagaggaagagcaaaacaaaagtaagaccaccgcac
agcaagcccgctgatcttcagacctggaggaggagatatgagggacattggagaagtgaattatataaatataaagtagtaaaaattgaacca
ttaggagtagcacccaccaaggcaaagagaagagtggtgcagagagaaaaaagagcagtgggaataggagctttgttccttgggttcttgg
gagcagcaggaagcactatgggcgcagcgtcaatgacgctgacggtacaggccagacaattattgtctggtatagtgcagcagcagaacaa
tttgctgagggctattgaggcgcaacagcatctgttgcaactcacagtctggggcatcaagcagctccaggcaagaatcctggctgtggaaag
atacctaaaggatcaacagctcctggggatttggggttgctctggaaaactcatttgcaccactgctgtgccttggaatgctagttggagtaataa
atctctggaacagatttggaatcacacgacctggatggagtgggacagagaaattaacaattacacaagcttaatacactccttaattgaagaat
cgcaaaaccagcaagaaaagaatgaacaagaattattggaattagataaatgggcaagtttgtggaattggtttaacataacaaattggctgtg
gtatataaaattattcataatgatagtaggaggcttggtaggtttaagaatagtttttgctgtactttctatagtgaatagagttaggcagggatattc
accattatcgtttcagacccacctcccaaccccgaggggacccgacaggcccgaaggaatagaagaagaaggtggagagagagacagag
acagatccattcgattagtgaacggatctcgacggtatcgaaagcttgggattcgaatttaaaagaaaaggggggattggggggtacagtgca
ggggaaagaatagtagacataatagcaacagacatacaaactaaagaactacaaaaacaaattacaaaaattcaaaattttcgggtttttcgaa
cctagggttccgcgttacataacttacggtaaatggcccgcctggctgaccgcccaacgacccccgcccattgacgtcaataatgacgtatgtt
cccatagtaacgccaatagggactttccattgacgtcaatgggtggagtatttacggtaaactgcccacttggcagtacatcaagtgtatcatatg
ccaagtacgccccctattgacgtcaatgacggtaaatggcccgcctggcattatgcccagtacatgaccttatgggactttcctacttggcagta
catctacgtttagtcatcgctattaccatggtgatgcggttttggcagtacatcaatgggcgtggatagcggtttgactcacggggatttccaagt
ctccaccccattgacgtcaatgggagtttgttttggcaccaaaatcaacgggactttccaaaatgtcgtaacaactccgccccattgacgcaaat
gggcggtaggcgtgtacggtgggaggtctatataagcagagctcgtttagtgaaccgtcagatcgcctggagacgccatccacgctgttttga
cctccatagaagaaccgagtttaaactccctatcagtgatagagatctccctatcagtgatagagagctagccccgggatcgatcaattgccgg
tcgccaccatggtgagcaagggcgaggagctgttcaccggggtggtgcccatcctggtcgagctggacggcgacgtaaacggccacaagt
tcagcgtgtccggcgagggcgagggcgatgccacctacggcaagctgaccctgaagttcatctgcaccaccggcaagctgcccgtgccct
ggcccaccctcgtgaccaccctgacctacggcgtgcagtgcttcagccgctaccccgaccacatgaagcagcacgacttcttcaagtccgcc
atgcccgaaggctacgtccaggagcgcaccatcttcttcaaggacgacggcaactacaagacccgcgccgaggtgaagttcgagggcgac
accctggtgaaccgcatcgagctgaagggcatcgacttcaaggaggacggcaacatcctggggcacaagctggagtacaactacaacagc
cacaacgtctatatcatggccgacaagcagaagaacggcatcaaggtgaacttcaagatccgccacaacatcgaggacggcagcgtgcag
ctcgccgaccactaccagcagaacacccccatcggcgacggccccgtgctgctgcccgacaaccactacctgagcacccagtccgccctg

agcaaagaccccaacgagaagcgcgatcacatggtcctgctggagttcgtgaccgccgccgggatcactctcggcatggacgagctgtac
aagtaaagtacgtaggtaccccagtgtggtggcctgcaggtgaattcactagtaccggtaggcctgtcgacgatatcgggcccgcggccgct
ggatcctctagactgcagctcgagtacccatacgacgtcccagactacgcttgagtttaaacacgcgtggtgtggaaagtccccaggctcccc
agcaggcagaagtatgcaaagcatgcatctcaattagtcagcaaccaggtgtggaaagtccccaggctccccagcaggcagaagtatgcaa
agcatgcatctcaattagtcagcaaccatagtcccgcccctaactccgcccatcccgcccctaactccgcccagttccgcccattctccgcccc
atggctgactaattttttttatttatgcagaggccgaggccgcctcggcctctgagctattccagaagtagtgaggaggcttttttggaggccatg
accgagtacaagcccacggtgcgcctcgccacccgcgacgacgtccctcgggccgtacgcaccctcgccgccgcgttcgccgactaccc
cgccacgcgccacaccgtggacccggaccgccacatcgagcgggtcaccgagctgcaagaactcttcctcacgcgcgtcgggctcgacat
cggcaaggtgtgggtcgcggacgacggcgccgcggtggcggtctggaccacgccggagagcgtcgaagcgggggcggtgttcgccga
gatcggcccgcgcatggccgagttgagcggttcccggctggccgcgcagcaacagatggaagggctcctggcgccgcaccggcccaag
gagcccgcgtggttcctggccaccgtcggcgtctcgcccgaccaccagggcaagggtctgggcagcgccgtcgtgctccccggagtgga
ggcggccgagcgcgccggggtgcccgccttcctggagacctccgcgccccgcaacctccccttctacgagcggctcggcttcaccgtcac
cgccgacgtcgaggtgcccgaaggaccgcgcacctggtgcatgacccgcaagcccggtgcctgaacgcgttccggaaatcaacctctgga
ttacaaaatttgtgaaagattgactggtattcttaactatgttgctccttttacgctatgtggatacgctgctttaatgcctttgtatcatgctattgcttc
ccgtatggctttcattttctcctccttgtataaatcctggttgctgtctctttatgaggagttgtggcccgttgtcaggcaacgtggcgtggtgtgcac
tgtgtttgctgacgcaacccccactggttggggcattgccaccacctgtcagctcctttccgggactttcgctttccccctccctattgccacggc
ggaactcatcgccgcctgccttgcccgctgctggacaggggctcggctgttgggcactgacaattccgtggtgttgtcggggaagctgacgt
cctttccatggctgctcgcctgtgttgccacctggattctgcgcgggacgtccttctgctacgtcccttcggccctcaatccagcggaccttcctt
cccgcggcctgctgccggctctgcggcctcttccgcgtctcgccttcgccctcagacgagtcggatctccctttgggccgcctccccgcctgt
ccggatggaagggctaattcactcccaacgaatacaagatctgctttttgcttgtactgggtctctctggttagaccagatctgagcctgggagct
ctctggctaactagggaacccactgcttaagcctcaataaagcttgccttgagtgcttcaagtagtgtgtgcccgtctgttgtgtgactctggtaa
ctagagatccctcagacccttttagtcagtgtggaaaatctctagcagtagtagttcatgtcatcttattattcagtatttataacttgcaaagaaatg
aatatcagagagtgagaggaacttgtttattgcagcttataatggttacaaataaagcaatagcatcacaaatttcacaaataaagcatttttttcac
tgcattctagttgtggtttgtccaaactcatcaatgtatcttatcatgtctggcatctatgtcgggtgcggagaaagaggtaatgaaatggcattatg
ggtattatgggtctgcattaatgaatcggccaacgatcccggtgtgaaataccgcacagatgcgtaaggagaaaataccgcatcaggcgctct
tccgcttcctcgctcactgactcgctgcgctcggtcgttcggctgcggcgagcggtatcagctcactcaaaggcggtaatacggttatccacag
aatcaggggataacgcaggaaagaacatgtgagcaaaaggccagcaaaaggccaggaaccgtaaaaaggccgcgttgctggcgtttttcc
ataggctccgcccccctgacgagcatcacaaaaatcgacgctcaagtcagaggtggcgaaacccgacaggactataaagataccaggcgtt
tccccctggaagctccctcgtgcgctctcctgttccgaccctgccgcttaccggatacctgtccgcctttctcccttcgggaagcgtggcgctttc
tcatagctcacgctgtaggtatctcagttcggtgtaggtcgttcgctccaagctgggctgtgtgcacgaaccccccgttcagcccgaccgctgc
gccttatccggtaactatcgtcttgagtccaacccggtaagacacgacttatcgccactggcagcagccactggtaacaggattagcagagcg
aggtatgtaggcggtgctacagagttcttgaagtggtggcctaactacggctacactagaaggacagtatttggtatctgcgctctgctgaagc
cagttaccttcggaaaaagagttggtagctcttgatccggcaaacaaaccaccgctggtagcggtggtttttttgtttgcaagcagcagattacg
cgcagaaaaaaaggatctcaagaagatcctttgatcttttctacggggtctgacgctcagtggaacgaaaactcacgttaagggattttggtcat
gagattatcaaaaaggatcttcacctagatccttttaaattaaaaatgaagttttaaatcaatctaaagtatatatgagtaaacttggtctgacagtta
ccaatgcttaatcagtgaggcacctatctcagcgatctgtctatttcgttcatccatagttgcctgactccccgtcgtgtagataactacgatacgg
gagggcttaccatctggccccagtgctgcaatgataccgcgagacccacgctcaccggctccagatttatcagcaataaaccagccagccg
gaagggccgagcgcagaagtggtcctgcaactttatccgcctccatccagtctattaattgttgccgggaagctagagtaagtagttcgccagt
taatagtttgcgcaacgttgttgaaaaaggatcttcacctagatccttttcacgtagaaagccagtccgcagaaacggtgctgaccccggatga
atgtcagctactgggctatctggacaagggaaaacgcaagcgcaaagagaaagcaggtagcttgcagtgggcttacatggcgatagctaga
ctgggcggttttatggacagcaagcgaaccggaattgccagctggggcgccctctggtaaggttgggaagccctgcaaagtaaactggatg
gctttctcgccgccaaggatctgatggcgcaggggatcaagctctgatcaagagacaggatgaggatcgtttcgcatgattgaacaagatgg
attgcacgcaggttctccggccgcttgggtggagaggctattcggctatgactgggcacaacagacaatcggctgctctgatgccgccgtgtt
ccggctgtcagcgcaggggcgcccggttctttttgtcaagaccgacctgtccggtgccctgaatgaactgcaagacgaggcagcgcggctat

cgtggctggccacgacgggcgttccttgcgcagctgtgctcgacgttgtcactgaagcgggaagggactggctgctattgggcgaagtgcc
ggggcaggatctcctgtcatctcaccttgctcctgccgagaaagtatccatcatggctgatgcaatgcggcggctgcatacgcttgatccggct
acctgcccattcgaccaccaagcgaaacatcgcatcgagcgagcacgtactcggatggaagccggtcttgtcgatcaggatgatctggacg
aagagcatcaggggctcgcgccagccgaactgttcgccaggctcaaggcgagcatgcccgacggcgaggatctcgtcgtgacccatggc
gatgcctgcttgccgaatatcatggtggaaaatggccgcttttctggattcatcgactgtggccggctgggtgtggcggaccgctatcaggaca
tagcgttggctacccgtgatattgctgaagagcttggcggcgaatgggctgaccgcttcctcgtgctttacggtatcgccgctcccgattcgca
gcgcatcgccttctatcgccttcttgacgagttcttctgaattttgttaaaatttttgttaaatcagctcatttttaaccaataggccgaaatcggcaa
catcccttataaatcaaaagaatagaccgcgatagggttgagtgttgttccagtttggaacaagagtccactattaaagaacgtggactccaacg
tcaaagggcgaaaaaccgtctatcagggcgatggcccactacgtgaaccatcacccaaatcaagttttttgcggtcgaggtgccgtaaagctc
taaatcggaaccctaaagggagcccccgatttagagcttgacggggaaagccggcgaacgtggcgagaaaggaagggaagaaagcgaa
aggagcgggcgctagggcgctggcaagtgtagcggtcacgctgcgcgtaaccaccacacccgcgcgcttaatgcgccgctacagggcgc
gtccattcgccattcaggatcgaattaattcttaattaacatcatcaataatatacctt
